# Evaluation of the Effects of Microgravity on Activated Primary Human Hepatic Stellate Cells

**DOI:** 10.3390/ijms23137429

**Published:** 2022-07-04

**Authors:** Koichi Fujisawa, Yuto Nishimura, Akino Sakuragi, Jolien Duponselle, Toshihiko Matsumoto, Naoki Yamamoto, Tomoaki Murata, Isao Sakaida, Taro Takami

**Affiliations:** 1 Department of Gastroenterology and Hepatology, Graduate School of Medicine, Yamaguchi University, Minami Kogushi 1-1-1, Ube 755-8505, Japan; fujisawa@yamaguchi-u.ac.jp (K.F.); g022ed@yamaguchi-u.ac.jp (Y.N.); v037eb@yamaguchi-u.ac.jp (A.S.); tm0831@yamaguchi-u.ac.jp (T.M.); sakaida@yamaguchi-u.ac.jp (I.S.); 2 Department of Environmental Oncology, Institute of Industrial Ecological Sciences, University of Occupational and Environmental Health, 1-1 Iseigaoka, Yahatanishi-ku, Kitakyushu 807-8555, Japan; 3 Departement of Dermatology, University Hospital of Ghent, C. Heymanslaan 10, 9000 Ghent, Belgium; jolienduponselle@gmail.com; 4 Health Administration Center, Yamaguchi University, Minami Kogushi 1-1-1, Ube 755-0046, Yamaguchi, Japan; nao-yama@yamaguchi-u.ac.jp; 5 Institute of Laboratory Animals, Science Research Center, Yamaguchi University, Yamaguchi 755-8505, Japan; ceb@yamaguchi-u.ac.jp

**Keywords:** hepatic stellate cell, microgravity, transcriptome

## Abstract

In recent years, research has been conducted to develop new medical treatments by simulating environments existing in space, such as zero-gravity. In this study, we evaluated the cell proliferation and gene expression of activated primary human hepatic stellate cells (HHSteCs) under simulated microgravity (SMG). Under SMG, cell proliferation was slower than in 1 G, and the evaluation of gene expression changes on day 1 of SMG by serial analysis of gene expression revealed the presence of Sirtuin, EIF2 signaling, hippo signaling, and epithelial adherence junction signaling. Moreover, reactive oxygen species were upregulated under SMG, and when N-acetyl-cystein was added, no difference in proliferation between SMG and 1 G was observed, suggesting that the oxidative stress generated by mitochondrial dysfunction caused a decrease in proliferation. Upstream regulators such as smad3, NFkB, and FN were activated, and cell-permeable inhibitors such as Ly294002 and U0126 were inhibited. Immunohistochemistry performed to evaluate cytoskeletal changes showed that more β-actin was localized in the cortical layer under SMG.

## 1. Introduction

Research to maintain the health of astronauts staying in zero-gravity for long periods of time, such as on the International Space Station (ISS), aims to develop novel techniques for the prevention, diagnosis, and treatment of diseases in space environments. Stem cell cultures in zero-gravity are able to maintain stem cell multifunctionality [1], and spaceflight exposure decreases proliferation and lineage restriction in mesenchymal stem cells (MSCs) [2]. Since it is often impracticable to study gravity in space, a simulated microgravity (SMG) apparatus (clinostat) has been developed that changes the direction of the gravity vector acting on the sample by rotating the specimen around two axes, canceling the effect of gravity as a time average [3]. The SMG environment is effective in promoting the regeneration of large organs [4] and causes changes in DNA methylation in myogenesis when MSCs are cultured under SMG [5]. Bone loss occurs in microgravity due to an imbalance between osteogenesis and osteoclasty, resulting in rapid bone loss. Mouse livers subjected to 13 days of spaceflight exhibited early signs of nonalcoholic fatty liver disease (NAFLD). However, due to the short exposure period, there was no collagen deposition and no overexpression of α-smooth muscle actin (α-SMA), while the gene expression of several hepatic stellate cell (HSC) activation markers increased [6].

Hepatocytes can be made to float freely in a culture medium under SMG and to slowly assemble to form three-dimensional (3D) tissue to minimize physical damage to cells and to promote the regeneration of bile ducts and blood vessels present in a healthy liver [7]. The differentiation of MSCs cultured under SMG is suppressed, while the differentiation of cells in hypergravity is promoted [8], and it is thus imperative to study the effect of gravity on various cells in detail. We previously analyzed gene expression in medaka fish raised on the Japanese Experiment Module of the ISS (dubbed “Kibo”) for two months using RNA extracted from whole liver [9], and this study involved further analysis of HSCs that play a key role in liver fibrosis.

HSCs are connective tissue cells within the lobule with a myofibroblast-like or lipid cell phenotype and are involved in the homeostasis, repair, and regeneration of the liver’s extracellular matrix (ECM), fibrosis, and the regulation of retinol metabolism, storage, and release [10]. After liver injury, HSCs transform into myofibroblast-like cells, which are the major source of type I collagen in fibrotic livers. HSCs act as regulators of the liver microenvironment through cell contraction and may cause intrahepatic portal hypertension [11,12]. The proliferation and migration of HSCs and chemokines are involved in liver inflammation and fibrosis, and new insights into the regulatory mechanisms of HSC activation hold the potential to reduce morbidity and mortality in patients with chronic liver injury. However, there have been no detailed reports to date discussing the effect of gravity on HSCs. In this study, we aimed to examine the effect of microgravity on liver fibrosis. We analyzed the effects of SMG on the gene expression and proliferation of activated primary human hepatic stellate cells (HHSteCs).

## 2. Results

### 2.1. HHSteC Proliferation Is Reduced under SMG

**Figure 1:**
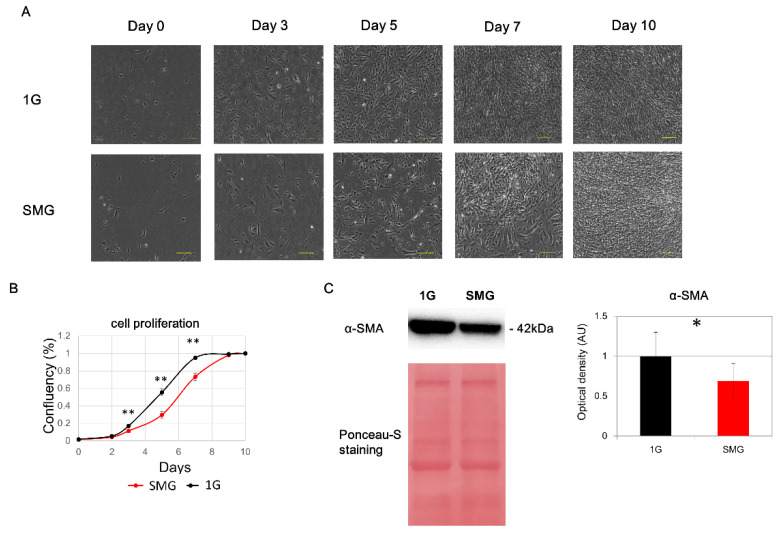
Evaluation of HHSteC proliferation under simulated microgravity (SMG). (**A**) Micrograph of cells under SMG (*n* = 6). (**B**) Evaluation of proliferation under SMG. Red line: SMG; black line: 1 G. (**C**) Western blotting analysis of α-SMA (α-smooth muscle actin), a stellate cell activation marker, and Ponceau-S staining as a loading control. The quantified bands were normalized to Ponceau (total protein) (*n* = 6). Statistical analysis was performed with Student’s *t*-test; * indicates *p* < 0.05, and ** indicates *p* < 0.01.

The proliferation of HHSteCs under SMG was evaluated using an artificial microgravity simulator (clinostat). No distinct morphological changes in either SMG or 1 G cell cultures were observed (Figure 1A). The confluency on days 3, 5, and 7 under SMG was significantly lower (*p* < 0.01) than that under 1 G at the same time points (Figure 1B). Protein expression of α-SMA, a stellate cell activation marker, was downregulated under SMG on day 2 (Figure 1C). 

### 2.2. Changes in Gene Expression Due to SMG Exposure

**Figure 2:**
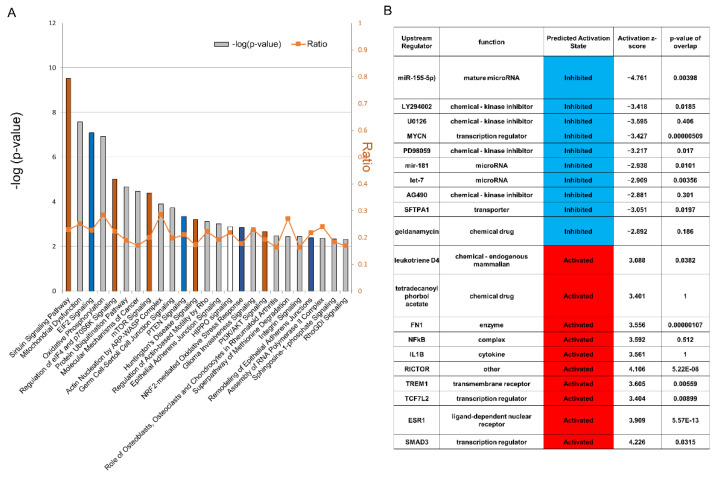
Gene expression (SMG vs. 1 G on day 1) by ingenuity pathway analysis (IPA) (*n* = 3). (**A**) Significantly enriched canonical pathways identified by IPA. The diagram shows significantly overrepresented canonical pathways. A multiple-testing corrected *p*-value was calculated using the Benjamini–Hochberg method to control the rate of false discoveries in statistical hypothesis testing. The ratio represents the number of molecules in a given pathway that meet cut criteria, divided by the total number of molecules that belong to the function. (**B**) Upstream regulators of genes with altered expression under simulated microgravity (SMG). The top 10 upstream regulators that were repressed or activated are shown. Blue: repressed regulators; red: activated regulators.

Serial analysis of gene expression (SAGE) was performed to investigate microgravity-induced changes in gene expression. We chose day 1 cells to evaluate early gene expression changes. Next, ingenuity pathway analysis (IPA) was performed for day 1 cells under SMG, which revealed the impact of SMG on canonical pathways, including Sirtuin, EIF2 signaling, mitochondrial dysfunction, hippo signaling, epithelial adhesion junction signaling, and phosphoinositide 3-kinase (PI3K) signaling (Figure 2A). The identified upstream regulators include smad3, NFkB, and fibronectin (FN). Ly294002, a specific inhibitor of cell-permeable phosphatidylinositol 3-kinase (PI3-kinase); U0126, involved in anoikis; and Myc and microRNAs such as miR-155-5p, miR-181, and let7 were inhibited (Figure 2B). The target molecules in the dataset are listed in Supplementary Table S1.

### 2.3. Mitochondrial Dysfunction Increases Oxidative Stress

**Figure 3:**
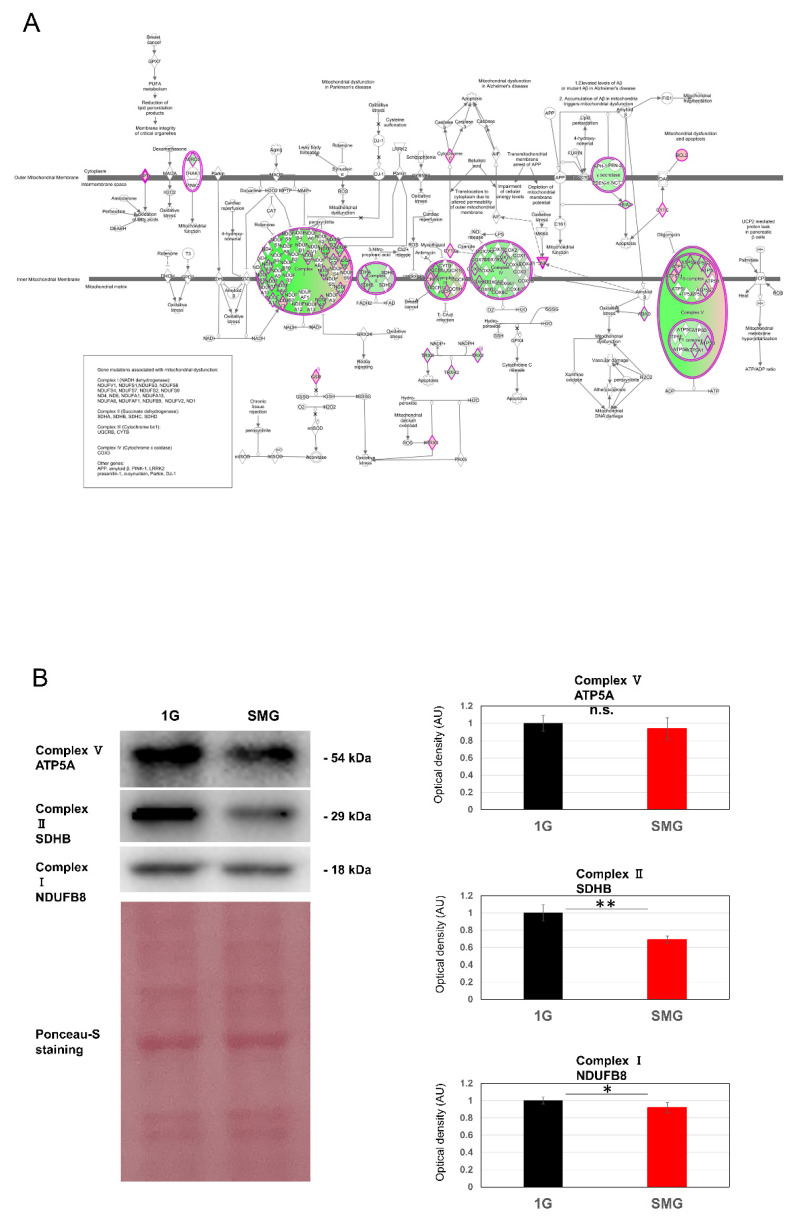
(**A**) Pathways related to mitochondrial dysfunction. Illustration of altered genes involved in IPA canonical pathway “mitochondrial dysfunction”. Overlap *p*-values, a measure of the overlap between observed gene expression changes and known targets regulated by transcriptional regulators, were calculated using Fisher’s exact test. Activation z-score (z-score), an indicator of the regulatory direction of a pathway, was calculated based on a database of molecular networks representing experimentally observed gene expression or transcriptional events, where a positive z-score means “activation” and a negative z-score means “repression”. In the mitochondrial dysfunction-pathway map obtained by IPA [13], upregulated components of gene expression involved in mitochondrial Complexes I-V are in red, and downregulated components are in green. (**B**) Western blotting analysis of ATP synthase lipid-binding protein, ATP5A (Complex V), succinate dehydrogenase, SDHB (Complex II), NADH dehydrogenase [ubiquinone] 1 beta subcomplex subunit 8, NDUFB8 (Complex I), and Ponceau-S staining as a loading control. The quantified bands were normalized to Ponceau (total protein) (*n* = 6). Statistical analysis was performed with Student’s *t*-test; * indicates *p* < 0.05, ** indicates *p* < 0.01, and n.s. indicates not significant.

**Figure 4:**
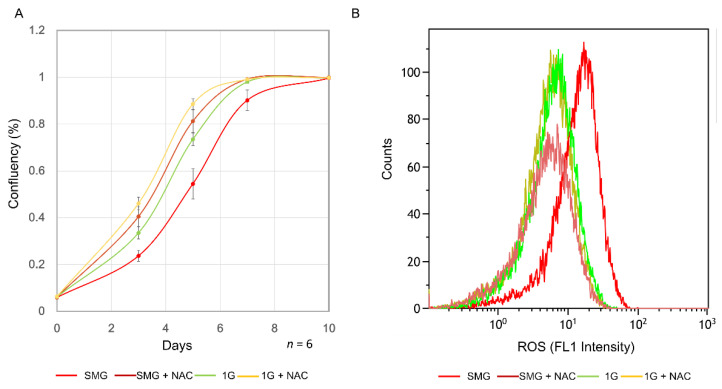
Evaluation of changes in oxidative stress due to simulated microgravity (SMG) exposure. (**A**) Changes in cell proliferation resulting from addition of 5 mM N-acetyl-cystein (NAC). (**B**) Evaluation of reactive oxygen species (ROS) (day 4). ROS activity was analyzed by flow cytometry at FL1 (490/525 nm) using a flow cytometer.

Evaluation of mitochondrial dysfunction among canonical pathways revealed changes in gene expression of Complexes 1–5 (Figure 3A). The genes in Figure 3 and their functions are listed in Supplementary Table S2. The protein expression of genes involved in mitochondrial complexes was evaluated (Figure 3B). Reactive oxygen species (ROS) increased under SMG (Figure 4A). To evaluate the relation between mitochondrial dysfunction and the inhibition of SMG-induced proliferation, N-acetyl-cystein (NAC) was added. As a result, inhibition of SMG-induced proliferation was reduced, suggesting that ROS inhibited proliferation (Figure 4B).

### 2.4. Effect of SMG on the Cytoskeleton

**Figure 5:**
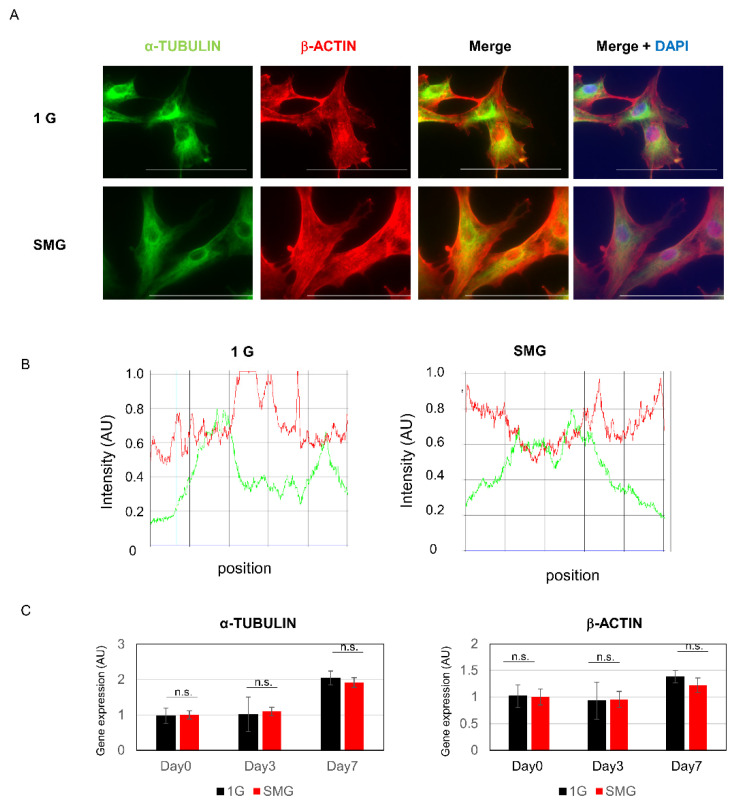
Cytoskeletal changes caused by SMG. (**A**) HHSteCs were cultured under SMG for 7 days. Green:α-tublin; red:β-actin; blue: DAPI staining; white line indicates 100 µm. (**B**) Intensity profiles of α-tubulin (green) and β-actin (red) along the line segment. (**C**) Gene expression analysis by real-time RT-PCR (*n* = 6). n.s. indicates not significant in statistical analysis.

To evaluate the effect of SMG on the HHSteC cytoskeleton, we used fluorescence immunohistochemistry using anti-β-actin and α-tubulin antibodies. While no significant change in the localization of α-tubulin was observed, a greater quantity of β-actin was localized in the cortical layer (Figure 5).

## 3. Discussion

Previous studies have shown that SMG produced by a clinostat can be used as a substitute for weightlessness in space in studies on myoblast differentiation [5]. The MSC microenvironment is garnering greater attention, as it can help maintain these cells in an undifferentiated state [14]. On the other hand, various studies on smooth muscle cells have been conducted to date and have yielded tremendous results [15,16,17,18,19]. HHSteCs, primary human hepatic stellate cells, showed decreased proliferation under SMG. In rat aortic smooth muscle cells, decreased proliferation and increased apoptosis were observed under SMG [20], indicating that decreased proliferation under SMG may be due to decreased expression of Akt, EGF, and bFGF [21,22]. Although one report showed reduced proliferation of umbilical cord-derived MSCs [23], another stated that the proliferation of bone marrow-derived MSCs markedly increased [24]. Further research is needed to better understand the changes in cell proliferation depending on the cell type.

This study evaluated the effects of SMG by comprehensive gene expression analysis. IPA suggested that the sirt pathway, mitochondrial dysfunction, and oxidative phosphorylation are involved. It has been reported that when human primary osteoblasts are exposed to SMG, prominent dysregulation of mitochondrion homeostasis occurs, and the mitochondrial energy potential, and hence that of the cell, changes [25]. It has also been reported that Sirtuin is involved in the regulation of mitochondrial metabolism [26] and of multiple metabolic pathways, such as lipid and glucose metabolism, ketone body synthesis, the urea cycle, and insulin secretion and is known to regulate energy homeostasis [27]. Although it is known that microgravity induces oxidative stress [28], the detailed mechanism has yet to be revealed [29]. However, it is interesting to note that when human Hodgkin’s lymphoma (HL) cells are cultured under SMG, ROS production and NADPH oxidase family gene expression increase, and autophagy due to mitochondrial dysfunction is enhanced [30], attracting attention to the relationship between SMG and mitochondria. In the present study, an increase in ROS was also observed under SMG, which may be one of the causes of the inhibition of cell proliferation. Further, when cells were cultured with NAC, which is known to cause growth inhibition and apoptosis at higher doses but to promote cell growth at lower doses [31], the difference in growth between 1 G and SMG disappeared, supporting that ROS is involved in growth under SMG. The PI3K/Akt pathway regulates cell survival, proliferation, and motility and is known to be involved in endothelial NO synthase production and the inhibition of apoptosis in cells exposed to SMG [32]. It has also been shown that p-AKT and p-PI3K increase under SMG and are involved in survival [33]. A novel regulatory mechanism of glycolysis through cytoskeletal regulation by PI3K [34] may be involved in the alteration of glycolysis due to mitochondrial dysfunction under SMG.

While microRNAs such as miR-155-5p, miR-181, and let7 have been raised as repressed upstream regulators, they are involved in the regulation of gene expression, such as in activation in HSCs, and are important as predictive and therapeutic markers of liver fibrosis [35]. Among the miRNAs, mir223 is involved in the effects of gravity on the liver, where long-term spaceflight affects many organs, increasing AST and ALT and inhibiting cell proliferation [36]. Moreover, let7 is known to be a tumor-suppressing miRNA and to decrease in fibroblasts in spaceflight [37]. It is known that the EMT and ERK1 pathways are activated when miR-155-5p is inhibited and that forced miR-155 expression markedly decreases mesenchymal markers and phosphorylated ERK1 expression and enhances E-cadherin expression, leading to the synchronous inhibition of EMT and ERK1 pathways and inducing HSC apoptosis [38,39]. Moreover, reduced miR-155 is associated with EMT and ERK1 pathway activation in hepatic fibrogenesis. Other reports have suggested that miR-155-5p is associated with aging [40] and that miR-181b promotes HSC proliferation by targeting p27. Overexpression of miR-181b increases the expression of α-SMA and type I collagen involved in the PTEN/Akt pathway and also induces cell proliferation, suggesting a profibrotic function [41]. U0126 has been shown to render MDA-MB231 and HBC4 sensitive to anoikis, and treatment with U0126 deprives cells of their scaffold to proceed to apoptosis. Although Smad3 had the highest activation z-score, it has been reported that Smad3 plays an important role in the morphological and functional maturation of hepatic myofibroblasts and is also involved in the regulation of cytoskeletal organization. Furthermore, it is interesting to note that Smad3-mediated signaling, the TGF-β pathway, has been suggested to play an important role in the morphological and functional maturation of hepatic myofibroblasts [42].

Changes in the cytoskeleton are known to occur in zero-gravity [43,44]. In particular, actin microfilaments, known to be the intracellular skeleton, are believed to mediate signal transduction occurring within cells. The quantity of F-actin decreases in A431 cells exposed to microgravity, suggesting that actin microfilaments are involved in gravity-sensitive signaling [45]. The present study also noted an accumulation of actin at the cell border but no clear change in tubulin levels. Research has revealed that the structure of the actin network reduces in width or in the amount of stress fibers in zero-gravity [46], in terms of distribution around the nucleus [47], and in terms of accumulation at the cell border [48]; by contrast, the reported changes in tubulin include thicker filaments [8], loss of structure, perinuclear clustering, or no clear effect [49], and changes in the amount of tubulin that were not constant [50].

In this study, we used activated primary human hepatic stellate cells. It is known that there are many differences between quiescent and activated HSCs. The effects of microgravity on liver fibrosis seem limited in the monoculture of HHSteCs. There is a possibility that damage to hepatocytes activates HHSteCs, so co-culturing with hepatocyte or vascular endothelial cells is necessary in future studies. It is also necessary to evaluate the effect of microgravity on quiescent HSCs by using three-dimensional cultures in a future study.

## 4. Materials and Methods

### 4.1. Cells and Cell Culturing

**Figure 6:**
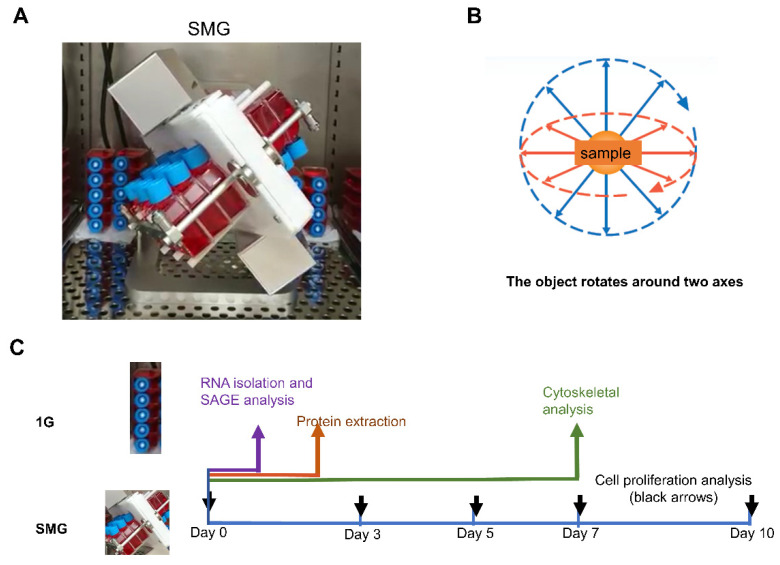
(**A**) By rotating the sample in three dimensions, gravity is evenly distributed, creating a simulated microgravity environment (https://prod.kiw.co.jp/medical/zeromo.html) (accessed on 23 December 2020). The gravity of all flasks was less than 10^−3^ G. (**B**) Schematic diagram of the clinostat. (**C**) A schematic representation of the experimental design. Black arrows indicate the day for cell proliferation analysis, a brown arrow indicates the day for protein extraction, a blue arrow indicates the day for RNA sampling, and a green arrow indicates the day for cytoskeletal analysis.

HHSteCs were purchased from ScienCell Research Laboratories, Inc. (catalog number 5300), and cells from passages 3–5 were used [51]. HHSteCs were cultured in Stellate Cell Medium (ScienCell, #5301) or Dulbecco’s Modified Eagle’s Medium (DMEM) (GIBCO, Grand Island, NY, USA) supplemented with 10% fetal bovine serum (FBS) (GIBCO) and penicillin/streptomycin (GIBCO) in a 12.5 cm^2^ culture flask (Falcon 353107) with an oxygen-permeable cap. Cells were seeded at a density of 500–1000 cells/cm^2^. The cells were cultured in a 3D clinostat (Zeromo CL5000, Kitagawa, Hiroshima, Japan) and kept in the same incubator as the 1 G control. The object is rotated around one axis perpendicular to the force of gravity. On the other hand, the object rotates around two axes to provide a status of vector averaged gravity in a 3D clinostat [52] (Figure 6).

### 4.2. Cell Proliferation Assay

Cells were seeded at a density of 500–1000 cells/cm^2^. The cells were cultured in a 3D clinostat for 7 days [53]. Cell proliferation was determined by measuring the area occupied by the cells using the NIKON imaging system (Biostudio-T, NIKON, Kanagawa, Japan). The clinostat was stopped for approximately 15 min to capture images. Cell proliferation was evaluated six times.

### 4.3. Total RNA Isolation

Total RNA was isolated from the cell using the RNeasy kit (Cat#74106, QIAGEN, Hilden, Germany) and purified according to the manufacturer’s instructions. RNA samples were quantified using an ND-1000 spectrophotometer (NanoDrop Technologies, Wilmington, DE, USA), and the quality was confirmed using the Experion System (Cat#7007106JA, Bio-Rad, Hercules, CA, USA).

### 4.4. Serial Analysis of Gene Expression (SAGE)

The Ion AmpliSeq Transcriptome Human Gene Expression Kit (A26326, Thermo Fisher, Tokyo, Japan) was used to construct a library. An Ion PI IC 200 Kit (4488985, ThermoFisher, Tokyo, Japan) and an Ion PI Chip Kit v2 BC were used for sequencing using an Ion Proton next-generation sequencer. Data were deposited in The Gene Expression Omnibus (GEO, http://www.ncbi.nlm.nih.gov/geo/) (accessed on 24 June 2021) as GSE178831 (direct link to deposited data: https://www.ncbi.nlm.nih.gov/geo/query/acc.cgi?acc=GSE178831) (accessed on 24 June 2021). Three samples in each group were evaluated in SAGE with no repetition. We determined significantly enriched canonical pathways and upstream regulators with Ingenuity Pathway Analysis (IPA) (Ingenuity Systems, INC. http://www.ingenuity.com) (accessed on 28 January 2021). The top 25 canonical pathways were assigned, and mitochondrial dysfunctions were evaluated in detail [13]. The top 10 activated upstream regulators and the top 10 inhibited upstream regulators are shown in the figure.

### 4.5. Real-Time RT-PCR

Total RNA was isolated from cells using Isogen (Life Technology, Tokyo, Japan) according to the manufacturer’s instructions. For cDNA synthesis, Taqman reverse transcription reagents (Roche Diagnostics, Indianapolis, IN, USA) were used as described in the manufacturer’s manual. Variations in the expression of the genes and control 18S ribosomal RNA were analyzed using a Step One Plus real-time PCR system (Life Technologies, Tokyo, Japan) with SYBR green. For the RT-PCR analysis, primers were chosen for their dissociation curves, lack of nonspecific amplification, and relatively good amplification efficiency. The base sequences for the utilized primers are as follows: beta-actin Forward 5′- CGGGACCTGACTGACTACCT -3′, Reverse 5′- CTCCTTAATGTCACGCACGA -3′; alpha-tubulin Forward 5′- CATTGAAAAGTTGTGGTCTGATCA -3′, Reverse 5′- GCTTGGGTCTGTAACAAAGCAT -3′; 18S rRNA Forward 5′- ACGGACAGGATTGACAGATTG -3′, Reverse 5′- ATCGCTCCACCAACTAAGAAC -3′.

### 4.6. Western Blot Analysis

Cell lysis buffer was prepared with 62.5 mM Tris-HCl (pH 6.8), 4% sodium dodecyl sulfate (SDS), and 200 mM dithiothreitol; electrophoresis was performed using a 12% acrylamide gel. For electrophoresis, we used 20 μg of sample protein. After subsequently transferring the proteins to polyvinylidene fluoride (PVDF) membranes and applying nonspecific epitope blocking using 5% skimmed milk, the following antibodies were applied overnight. Antibodies against α-SMA (#19245, Cell Signaling Technology, Tokyo, Japan) and against ATP5A, SDHB, and NDUFB8 (Total OXPHOS Human WB antibody Cocktail, ab110411, Abcam, Tokyo, Japan) were used. Ponceau-S stain was used as a loading control.

### 4.7. ROS Activity Evaluation

ROS activity was measured with the ROS-ID Total ROS detection kit (Enzo Life Sciences, Farmingdale, NY, USA) according to the manufacturer’s protocol. Briefly, cells were cultured for 4 days and collected. Subsequently, cells were washed and resuspended in ROS detection solution. Samples were incubated in the dark for 30 min and analyzed by flow cytometry at FL1 (490/525 nm) using a cytometer (Gallios, 3L10C, BECKMAN COULTER, Tokyo, Japan). The data were evaluated by triplicate measurements.

### 4.8. Fluorescent Immunostaining

Immunohistochemistry was performed according to established methods [54]. Briefly, liver tissues were fixed with Bouin’s solution, and paraffin-embedded sections, 3 μm thick, were prepared. After deparaffinization using lemosol and ethanol, endogenous peroxidase was blocked with fresh 0.3% hydrogen peroxide in methanol at room temperature for 30 min, followed by antigen activation at 95 °C for 6 min in 10 mM sodium citrate buffer (pH 6.0). The sections were incubated with normal goat serum (Vector Laboratories; Burlingame, CA, USA) for 20 min at room temperature and washed. Specimens were then incubated with 1:300-diluted antibodies (anti-α-tubulin antibody (CALBIOCHEM DM1A, San Diego, CA, USA), anti-β-actin antibody (Sigma, clone AC-74, St. Louis, MO, USA)) at 4 °C overnight in a moist chamber. After washing thrice with phosphate-buffered saline (PBS), sections were studied using the Opal 4-color manual IHC kit NEL81000KT (PerkinElmer, Waltham, MA, USA). The data were evaluated by triplicate measurements. Cells were observed using a Fluorescence Microscope Keyence BZ-9000 BIOREVO (Osaka, Japan) equipped with blue (λex 377 nm, λem 447 nm), green (λex 472 nm, λem 520 nm), and red (λex 543 nm, λem 593 nm) filters with the acquisition software Keyence BZ-II Viewer (Osaka, Japan).

### 4.9. Statistical Analysis

To estimate whether the obtained data follow a normal distribution or not, we performed the χ2-test using Statcel3 (OMS publishing Inc., Tokyo, Japan), an add-in software. All groups were normally distributed. Student’s *t*-test was performed for two groups with Statcel3.

## Data Availability

The data presented in this study are available on request from the corresponding author. SAGE data are deposited in The Gene Expression Omnibus (GEO, http://www.ncbi.nlm.nih.gov/geo/) (accessed on 24 June 2021) as GSE178831 (direct link to deposited data: https://www.ncbi.nlm.nih.gov/geo/query/acc.cgi?acc=GSE178831) (accessed on 24 June 2021).

## Supplementary Materials

The following supporting information can be downloaded at: https://www.mdpi.com/article/10.3390/ijms23137429/s1.
