# Supplementary material for: Evaluation of the Effects of Microgravity on Activated Primary Human Hepatic Stellate Cells
Source: Int J Mol Sci. 2022 Jul 4;23(13):7429. doi: 10.3390/ijms23137429 (PMC9266956; doi:10.3390/ijms23137429)
Supplement: Supplementary file 1 [file ijms-23-07429-s001.zip › ijms-1782331-supplementary.pdf]

Supplementary Table S1

The list of target molecules in dataset

Upstream Regulator	Target molecules in dataset
SMAD3	APOA1,BCL2,BCL2L11,CDH2,COL1A1,CTNNB1,DOCK4,EGR1,ELN,FST,GLI2,IL11,INPP5D,ITGB1,MBOAT2,MEF2D,MYC,PDGFB,PMEPA1,PTGS2,PTPRK,RUNX2,S1PR1,SERPINE1,SMAD5,TAGLN,TBX3,TGFB2,TIMP1,TIMP3,TNC,VEGFA,XIAP,ZYX
ESR1	ABCC3,ABHD2,ADAM17,ADAMTS13,AES,AHNAK,AKT1S1,ALCAM,ANAPC2,ANGPTL2,AP1S3,AP4M1,APOA1,ARHGEF11,ARRB1,ASPM,ATP1B1,BAX,BAZ2A,BCL2,BCL2L11,BCL6,BET1L,BRAP,BRCA2,BYSL,CALD1,CAMK1,CARM1,CASP10,CBL,CCND3,CCNH,CDC42SE2,CDC5L,CDCA8,CDK1,CDK5RAP2,CDK6,CEBPB,CENPA,CENPQ,CEP135,CHM,CLN3,COG2,COG6,COL13A1,COL5A2,COMT,COPA,CPE,CRADD,CRK,CSNK1A1,CTNNB1,CXCL8,CYP1A1,CYP1B1,DDB2,DDX17,DNAJC1,DNAJC21,DOCK4,DR1,DSCR3,DST,DTNA,DUSP11,EFEMP1,EGR1,EML1,ERGIC2,FASN,FOSB,FOXC1,FOXM1,FST,FZD2,GAL,GLI2,GNAQ,GOPC,GOSR1,GPAM,GREM1,GSN,HAX1,HLA-C,HMGA2,HNRNPD,HOMER3,HTRA2,IER3,IFITM1,IFITM3,IGFBP2,IGFBP4,IL6ST,IPO7,ITGB1BP1,JAK1,KHDRBS1,KIF3B,KLHL20,KLHL24,LAMB1,LIMA1,MAP3K13,MAP3K4,MAST2,MDM4,MEF2D,MND1,MPC1,MYC,NCAPG,NCOA3,NFAT5,NFKBIA,NIPSNAP1,NUP205,NUP88,OPHN1,OSTF1,PAK2,PALLD,PCGF2,PDCD5,PGLS,PIAS4,PKN2,PLAUR,PLOD2,PMAIP1,PPM1B,PRKAA1,PRKACB,PRKX,PRSS23,PRUNE1,PSMD3,PTGS2,PTP4A2,PWP1,PYGL,RAB31,RBBP4,RBL2,RDX,RERE,RHOQ,RIPK1,ROBO1,RPS6KB1,RPS6KB2,RUNX2,SAMHD1,SCAMP1,SCARB2,SEC14L1,SEC31A,SEC63,SEMA3F,SERPINB9,SERPINE1,SFXN3,SH3GLB2,SHROOM1,SLC12A4,SLC25A36,SLC2A4RG,SLC3A2,SLC44A1,SLC7A11,SLC7A5,SLC9A8,SMAD2,SMAD5,SMAD6,SMURF1,SORD,SOX9,SP100,SPTAN1,SPTBN1,SQSTM1,STARD3,STC2,TACC1,TAX1BP1,TBRG1,TCF7L2,TEAD4,TESK1,TGFB2,THBS3,TIMM17B,TM4SF1,TMF1,TMOD3,TNPO1,TRIB3,TRIO,TSPAN9,TSPYL2,TXNIP,UBE2T,UBL3,ULBP2,VEGFA,VEZF1,VPS35,WEE1,ZNF107
TCF7L2	ACADL,ADGRG1,ANXA1,ARHGEF10,CHMP1B,CPD,CREB3L2,CTNNAL1,DEGS1,DHCR24,DHRS7,DOCK10,EPAS1,EPCAM,ERBIN,ERF,FAM91A1,FBXO8,GCLM,GLTP,GSN,ID4,ILK,IPO13,LAMC2,MYC,NCOR2,NDE1,NPAT,NPC1,OTUD7B,PHACTR4,PIP4K2A,PLAT,PPP1R14A,PTPN11,RAB31,RNF13,SLC22A23,SLC44A1,SMURF1,SNX18,STK17B,STK39,SYDE2,SYPL1,TBC1D14,TMEM141,TPPP,UBE2G

	1,VEGFA
TREM1	ADORA2B,ADRB2,ARRDC4,ASNS,ATP1B1,BCL2,CDK1,CEBPB,CXCL2,CXCL5,CXCL8,DTL,E2F7,EGR1,EIF2AK3,F3,FNDC3A,GCLM,GIPR,GREM1,IL1R1,ISG15,MFN1,MOAP1,MYD88,NEDD4L,NFKBIA,NPC1,NR4A2,NRIP3,PHLDA1,PTGS2,SNUPN,TFPI2,THBD,TLR4
RICTOR	ATP5A1,ATP5F1,ATP5G1,ATP5G2,ATP5G3,ATP5J,ATP5L,ATP5O,ATP6V0A2,ATP6V1C1,ATP6V1D,BAD,BAX,BCL2L11,COX6B1,DAXX,EGR1,ISG15,NDUFA11,NDUFA2,NDUFA4,NDUFA4L2,NDUFA7,NDUFB5,NDUFB6,NDUFB8,NDUFS3,NDUFS6,NDUFS7,NDUFV2,NDUFV3,PSMA6,PSMA7,PSMB8,PSMC2,PSMC3,PSMD13,PSMD2,PSMD3,PSMD8,PSME1,PTGS2,RPL26,RPL8,RPLP0,RPS10,RPS21,RPS23,RPS9,SDHA,SDHB,SEM1,SREBF1,UQCRB,UQCRC1,UQCRFS1
IL1B	ABCC3,ADAMTS1,ADAMTS5,ADORA2B,ADRB2,ANXA1,ARL6IP5,ASB7,BAX,BCL2,BMF,BTRC,CASP10,CD40,CEBPB,COL1A1,CTNNB1,CXCL2,CXCL5,CXCL6,CXCL8,CYP1A1,DCN,DNMT1,DPP4,DR1,EGLN1,EGR1,EIF4E,ELAVL1,ELN,ENPP1,EPAS1,F3,FOSB,FST,GAS6,GBP3,GCLC,HERC5,HMGA1,HSF1,IER3,IGFBP4,IL11,IL1R1,ISG15,ISL1,ITGAV,ITGB1,LAMC2,LDHA,LOX,MTDH,MYC,NAMPT,NFAT5,NFKBIA,NFKBIZ,NMI,NR4A2,NRP1,ODC1,P4HB,PDCD1LG2,PDE4B,PDGFB,PDGFRA,PHLDA1,PLA2G4A,PLAT,PLD3,POSTN,PPP1R14A,PSMB8,PTGES,PTGS2,RIPK2,RUNX2,S100A10,SDC1,SERPINB9,SERPINE1,SLC7A11,SOX9,SREBF1,TFPI2,THBD,TIMP1,TIMP3,TLR4,TXNIP,TYMP,UGCG,USP18,VASP,VEGFA,ZYX
NFkB (complex)	ADORA2B,AHR,ALDH1A3,B4GALT1,BAD,BAX,BCL2,BLVRA,CAPNS1,CCNH,CD40,CEBPB,CRADD,CTNNB1,CXCL2,CXCL5,CXCL6,CXCL8,CYLD,DAD1,DAXX,E2F7,EDNRB,EGR1,ELK1,ENPP1,EPAS1,EPCAM,F3,FOXF1,FST,GAS6,GCLC,GFPT2,GLI2,HERC5,IER3,IGFBP2,IRF3,ISG15,ISL1,ITGAV,ITGB1,KDM6B,MSH2,MTA1,MTSS1,MYC,NAMPT,NCOA7,NFKBIA,NFKBIZ,NUAK2,ODC1,PMAIP1,PTGES,PTGS2,RBPJ,RRAS,SERPINE1,SLC7A5,STK10,TCF12,TFPI2,TIMP1,TLR4,TPMT,TRIB3,VEGFA,XIAP,XRCC5,XRCC6
FN1	APLP2,BCL2,BNIP3L,CANX,CCT4,CDH2,CDK6,CEBPB,CLIC4,COL1A1,CXCL2,CXCL5,CXCL8,DHCR24,EIF2S3,F3,GDI2,GPX1,IL1R1,ITGAV,ITGB1,LGALS8,MYC,NFKBIA,NUP88,PDGFB,PDGFRA,PLAUR,PREB,RBBP4,RDX,RECK,RUNX2,SDC1,SEC23B,SERPINE1,SOX9,SQSTM1,SURF1,TAGLN,U2AF2,UBE4B,VPS45,VPS4A,XIAP,ZYX

phorbol myristate acetate	ADAM15,ADAM17,ADAMTS1,AHCTF1,ALDH1A3,AMIGO2,ANXA1,APRT,ATP2B4,BAX,BCL2,BNIP3L,C3orf52,CAPG,CCND3,CCNH,CDK1,CMPK1,CPE,CTNNB1,CUL3,CXCL2,CXCL8,CYP1A1,DEK,DNMT1,DTL,EDF1,EEF1D,EGR1,EIF2AK2,EIF4E,EIF4EBP2,ELK1,F3,FOSB,FOXP1,FST,GAL,GCLC,GNAI2,GSTM1,GSTP1,HMGA1,HMGA2,IGFBP2,IL11,ISG15,ITGA2,ITGB1,KCNN4,KHDC1,KLF2,KLF4,KLF6,LIN7A,LIPE,MEIS2,MRPL12,MYC,MYH9,NAB2,NCOA7,NFAT5,NFKBIA,NR4A2,NRP1,ODC1,PAK2,PDGFRB,PLAT,PLAUR,PLIN3,POLR1C,PPP1R3D,PRKX,PSMC3,PTGES,PTGS2,PTPRE,RAD17,RBBP4,RBPJ,RECQL4,RGS2,RPL19,RRP15,RUVBL2,RYBP,S100A10,SDC1,SEM1,SERPINB9,SERPINE1,SLC29A1,SLC7A11,SMAD6,SNCG,STK11,TEAD4,TFPI2,TIMP1,TLR4,TNFRSF12A,TSFM,TUFM,TYMP,UBXN4,UGCG,ULBP2,VEGFA,VLDLR
leukotriene D4	ADAMTS1,CXCL2,CXCL8,EGR1,F3,ITGB1,KLF2,KLF4,NR4A2,PDGFRB,PTGS2,RGS2
geldanamycin	BAX,BCL2,CD40,CDH24,CHUK,CXCL8,CYP1A1,EIF1AD,HSF1,MYC,PCDH1,PROSER3,PTGS2,QSOX1,RIPK1,RPS6KA2,THBD,TMEM110,TPMT,TPPP,WEE1
SFTPA1	ADRB2,COL1A1,CRLF1,CXCL2,CXCL5,CYP1B1,EGR1,FOSB,GFPT2,GSTO2,KCNMB4,KLF2,LOX,LRIG1,MYC,NAMPT,PHLDA1,SERPINE1,SH3PXD2A
AG490	BAX,BCL2,CCND3,CXCL8,MYC,PTGS2,S100A10,TAGLN,TIMP1,VEGFA
let-7	BMPR1A,BRCA2,CDC16,CDCA8,CDH2,CDK1,CDK6,COL1A1,CPED1,DBF4,GZF1,HMGA2,MCM3,MCM4,MCM5,MYC,MYD88,ORC1,PTGS2,RRM2,SMAD2,SMAD4,SOX9,TAB2,TLR4,ZNF512B
mir-181	ADCY9,ATG5,BCL2,BCL2L11,CYLD,KLF6,PBX3,PTPN11,RUNX2,SPTLC1,TIMP3
PD98059	ACAT2,AMD1,ANXA1,APOA1,ARRB1,BAX,BCL2,BCL2L11,BCL6,CD40,CDH2,CEBPB,COL1A1,CTNNB1,CTSK,CXCL2,CXCL5,CXCL8,CYCS,CYP1A1,CYP1B1,DNMT1,EDNRB,EEF1A1,EGR1,EIF2AK3,ELK1,ELN,EMP3,ENO2,EPAS1,F3,FABP4,FAH,FAM13A,FASN,FOSB,HMGA2,HPCAL1,IER3,IGF2BP2,ITGA2,ITGAV,ITGB1,LDHA,MYC,MYH4,NPC1,NXN,ODC1,PABPC1,PDGFB,PDGFRA,PHLDA1,PIK3R1,PLA2G4A,PLAT,PLAUR,POSTN,PRSS3,PTGES,PTGS2,RAB20,RECK,RRM2,RUNX2,S100A10,SDC1,SERPINE1,SIRPA,SLC3A2,SLC8A1,SMAD4,SNRPD1,SQLE,SREBF1,STOM,TCF3,TIMP1,TUBB2A,TXNIP,TYMP,VEGFA
MYCN	ABCA2,ABCB7,ABCC3,ABCC4,ABCF3,AEBP2,ARPC1B,BAX,CCNH,CHPF2,CITED2,CKAP4,COL1A1,COL5A2,DKK3,EEF1A1,EEF1D,EIF3C,HMGA1,IGFBP7,ITGA2,ITGB1,KDM5B,LDHA,MTF2,MYH9,NCL,ODC1,PMAIP1,PSMA7,RBBP4,RPL19,RPL26,RPL29,RPL31,RPL35,RPL37,RPL39,RPL8,RPLP0,RPS23,RPS25,RPS28,RPS4X,RPS7,RPS9,S100A10,SERPINE1,SORD,TAGLN,TPI1,TUFM,WAC,ZFAND5,ZYX

U0126	ADORA2B,BAD,BAX,BCL2,BCL2L11,BRAP,CCND3,CEBPB,COL1A1,CTNNB1,CXCL2,CXCL5,CXCL8,CYP1A1,CYP1B1,EDNRB,EEF1A1,EGR1,EIF2AK3,F3,FABP4,FOSB,GAMT,GCLC,GFPT2,GRAMD1B,GSN,GSTA4,IER3,ITGB1,ITPKC,KCNN4,KDM6B,MYC,NR4A2,NXPH4,PABPC1,PARP4,PHLDA1,PLA2G4A,PLAUR,POSTN,PTGS2,RUNX2,SERPINE1,SIRPA,SLC25A20,SOX9,STC2,STK17A,TCF7L2,TFPI2,TGFB2,TIMP3,TMEM184B,TOP3B,TYMP,VEGFA,WWTR1
LY294002	ACSM3,ADAM17,ADAMTS1,ADRB2,AGL,BAD,BAX,BCL2,BCL2L11,BCL6,BRAP,BRCA2,CCND3,CDK6,CDR1,CEBPB,COL1A1,CTNNB1,CUBN,CXCL2,CXCL5,CXCL6,CXCL8,DHCR24,EGR1,EMILIN1,EPAS1,F3,FASN,FER,FOSB,FOXC1,GCLC,GK,GSR,GSTA4,GSTM3,GSTP1,HMGB2,HSD17B6,IER3,IGFBP2,ITGAV,LAMC2,MAB21L1,MEIS2,MTA1,MYC,NDC80,NFATC3,NFKBIA,NPTX1,NR4A2,NTRK3,PDE4B,PGK1,PHLDA1,PIK3R1,PMAIP1,POSTN,PTGS2,PTPRU,RBL2,RGS2,RUNX2,S1PR1,SERPINE1,SPAG5,SQLE,SREBF1,TAGLN,TFPI2,THBD,TOP3B,TYMP,UBE2C,VEGFA,XIAP,ZNF230
miR-155-5p (miRNAs w/seed UAAUGCU)	AMIGO2,ARID2,ATP6V1C1,CEBPB,CHAF1A,CTNNB1,CXCL2,CXCL8,DHX40,INPP5D,LCLAT1,MEIS1,MSH2,MYD88,NARS,PIK3R1,PKN2,PMAIP1,PTGS2,RAB23,RIPK1,SCAMP1,SERPINE1,SLC30A1,SMAD2,SYPL1,TAB2,TCF7L2,WDFY1,WEE1

Supplementary Table S2

The list of genes related to mitochondrial dysfunction and their functions

Symbol	Entrez Gene Name	Entrez Gene	Expr Fold Change	Expr p-value	GO function
APH1A	aph-1 homolog A, gamma-secretase subunit	51107	-1.381	0.00106	endopeptidase activity
ATP5F1A	ATP synthase F1 subunit alpha	498	-1.285	0.00692	adenyl ribonucleotide binding
ATP5MC1	ATP synthase membrane subunit c locus 1	516	-2.687	0.0446	hydrogen ion transmembrane transporter activity activity
ATP5MC2	ATP synthase membrane subunit c locus 2	517	-1.39	0.0322	hydrogen ion transmembrane transporter activity
ATP5MC3	ATP synthase membrane subunit c locus 3	518	-1.869	0.0498	hydrogen ion transmembrane transporter activity
ATP5MG	ATP synthase membrane subunit g	10632	-1.316	0.0387	hydrogen-exporting ATPase activity
ATP5PB	ATP synthase peripheral stalk-membrane subunit b	515	1.542	0.038	ATPase activity
ATP5PF	ATP synthase peripheral stalk subunit F6	522	1.341	0.00258	ATPase activity
ATP5PO	ATP synthase peripheral stalk subunit OSCP	539	-1.649	0.0147	ATPase activity
BCL2	BCL2 apoptosis regulator	596	1.753	0.0357	BH3 domain binding
COX6B1	cytochrome c oxidase subunit 6B1	1340	-2.269	0.0418	cytochrome-c oxidase activity
COX6C	cytochrome c oxidase subunit 6C	1345	-1.7	0.0359	cytochrome-c oxidase activity
COX7C	cytochrome c oxidase subunit 7C	1350	-1.842	0.0474	cytochrome-c oxidase activity
CPT1A	carnitine palmitoyltransferase 1A	1374	1.467	0.0115	carnitine O-palmitoyltransferase activity
CYCS	cytochrome c, somatic	54205	1.334	0.0149	electron transporter
GSR	glutathione-disulfide reductase	2936	1.693	0.00259	electron carrier activity
HSD17B10	hydroxysteroid 17-beta dehydrogenase 10	3028	-1.701	0.0145	3-hydroxy-2-methylbutyryl-CoA dehydrogenase activity
HTRA2	HtrA serine peptidase 2	27429	-1.803	0.022	hydrolase activity
NDUFA2	NADH:ubiquinone oxidoreductase subunit A2	4695	-2.867	0.0443	NADH dehydrogenase (ubiquinone) activity
NDUFA4	NDUFA4 mitochondrial complex associated	4697	-1.812	0.00995	cytochrome-c oxidase activity
NDUFA7	NADH:ubiquinone oxidoreductase subunit A7	4701	-1.577	0.0177	NADH dehydrogenase (ubiquinone) activity
NDUFA11	NADH:ubiquinone oxidoreductase subunit A11	126328	-1.828	0.0246	NADH dehydrogenase (ubiquinone) activity
NDUFA4L2	NDUFA4 mitochondrial complex associated like 2	56901	-1.617	0.0283	cytochrome-c oxidase activity
NDUFB5	NADH:ubiquinone oxidoreductase subunit B5	4711	-1.462	0.0471	NADH dehydrogenase (ubiquinone) activity
NDUFB6	NADH:ubiquinone oxidoreductase subunit B6	4712	-2.081	0.0435	NADH dehydrogenase (ubiquinone) activity
NDUFB8	NADH:ubiquinone oxidoreductase subunit B8	4714	-2.871	0.0163	NADH dehydrogenase (ubiquinone) activity
NDUFB11	NADH:ubiquinone oxidoreductase subunit B11	54539	-1.503	0.0201	protein binding
NDUFS3	NADH:ubiquinone oxidoreductase core subunit S3	4722	-1.996	0.0276	electron carrier activity
NDUFS6	NADH:ubiquinone oxidoreductase subunit S6	4726	-3.257	0.049	electron carrier activity
NDUFS7	NADH:ubiquinone oxidoreductase core subunit S7	374291	-1.474	0.011	4 iron, 4 sulfur cluster binding
NDUFV2	NADH:ubiquinone oxidoreductase core subunit V2	4729	-2.297	0.0262	2 iron, 2 sulfur cluster binding
NDUFV3	NADH:ubiquinone oxidoreductase subunit V3	4731	1.629	0.0271	NADH dehydrogenase (ubiquinone) activity
PRDX3	peroxiredoxin 3	10935	1.357	0.00315	alkyl hydroperoxide reductase activity
RHOT2	ras homolog family member T2	89941	-1.404	0.0161	calcium ion binding
SDHA	succinate dehydrogenase complex flavoprotein subunit A	6389	-1.558	0.0437	electron carrier activity; flavin adenine dinucleotide binding
SDHB	succinate dehydrogenase complex iron sulfur subunit B	6390	-1.757	0.00446	2 iron, 2 sulfur cluster binding
SURF1	SURF1 cytochrome c oxidase assembly factor	6834	-1.455	0.0484	cytochrome-c oxidase activity
TXN2	thioredoxin 2	25828	-1.453	0.0303	peptide-methionine (R)-S-oxide reductase activity oxidoreductase activity
TXNRD2	thioredoxin reductase 2	10587	-1.32	0.0373	electron carrier activity; flavin adenine dinucleotide binding thioredoxin-disulfide reductase activity
UQCRB	ubiquinol-cytochrome c reductase binding protein	7381	-2.757	0.0244	protein binding
UQCRC1	ubiquinol-cytochrome c reductase core protein 1	7384	1.459	0.0234	catalytic activity
UQCRFS1	ubiquinol-cytochrome c reductase, Rieske iron-sulfur polypeptide	7386	1.349	0.0294	2 iron, 2 sulfur cluster binding
